# Growth and Cell Size of Microalga *Auxenochlorella protothecoides* AS-1 under Different Trophic Modes

**DOI:** 10.3390/microorganisms12040835

**Published:** 2024-04-20

**Authors:** Haoyu Chen, Ana Sosa, Feng Chen

**Affiliations:** 1 Institute of Marine & Environmental Technology, University of Maryland Center for Environmental Science, Baltimore, MD 21613, USA; hchen@umces.edu (H.C.); asosa@umces.edu (A.S.); 2 Maryland Sea Grant College, University of Maryland Center for Environmental Science, Cambridge, MD 21613, USA

**Keywords:** trophic mode, cell size, Coulter counter

## Abstract

Certain microalgal species can grow with different trophic strategies depending on the availability of nutrient resources. They can use the energy from light or an organic substrate, or both, and can therefore be called autotrophs, heterotrophs, or mixotrophs. We recently isolated a microalgal strain from the microplastic biofilm, which was identified as *Auxenochlorella protothecoides*, AS-1. Strain AS-1 grew rapidly in bacterial culture media and exhibited different growth rates and cell sizes under different trophic conditions. We compared the growth performance of AS-1 under the three different trophic modes. AS-1 reached a high biomass (>4 g/L) in 6 days under mixotrophic growth conditions with a few organic carbons as a substrate. In contrast, poor autotrophic growth was observed for AS-1. Different cell sizes, including daughter and mother cells, were observed under the different growth modes. We applied a Coulter Counter to measure the size distribution patterns of AS-1 under different trophic modes. We showed that the cell size distribution of AS-1 was affected by different growth modes. Compared to the auto-, hetero- and mixotrophic modes, AS-1 achieved higher biomass productivity by increasing cell number and cell size in the presence of organic substrate. The mechanisms and advantages of having more mother cells with organic substrates are still unclear and warrant further investigations. The work here provides the growth information of a newly isolated *A. protothecoides* AS-1 which will be beneficial to future downstream applications.

## 1. Introduction

Microalgae are important primary producers in the ecosystem. Microalgae have gained a lot of attention recently because they can produce valuable products that have variable applications (i.e., nutrient supplements, animal feeds, biofertilizers, cosmetics, renewable energy, etc.) [1,2,3]. To achieve high algal biomass, fast-growing algae are usually preferred for industrial processes. Understanding the growth performance of microalgae is essential to their biotechnological applications. Some microalgae can grow auto-, hetero-, or mixotrophically depending on the availability of inorganic and organic carbon [4,5,6]. For example, microalgae such as *Chlorella vulgaris* [7], *Dunaliella salina* [8], and *Scenedesmus obliquus* [9] are able to use carbon dioxide to make organic carbon through photosynthesis and can also assimilate organic carbon to support their growth. They can assimilate organic carbon for heterotrophic growth in dark environments and grow mixotrophically if additional light is provided.

Usually, the mitosis of eukaryotic microalgae happens as binary fission when a single cell (called the mother cell) is divided into two cells (called the daughter cell) [10,11,12]. However, among eukaryotic microalgae, another mechanism called multiple fission (or multi-fission) that produces more than two daughter cells (or autospores) can also happen [11,13,14,15]. In this case, mother cells may contain multiple daughter cells and increase in size before they release daughter cells [15,16]. Because of this nature, microalgal cells with different cell sizes can be produced under different nutrient environments [12]. It has been reported that the size structure of a microalgal population can be affected by its physiological status [17,18,19,20,21,22]. In reality, a microalgal culture consists of cells in different cell cycles with different sizes. Solely considering the population as having individual cells with the same growth rate, biochemical composition, and metabolism cannot reflect the situation behind growth [23]. Therefore, characterizing growth and cell size distribution will deepen our understanding of the growth performance of microalgae in different environments.

Microscopic observation is a traditional way to observe cell morphology and quantify cell number. When many samples are involved, counting cells, and measuring their size using microscopes can be tedious and time-consuming. Moreover, the complexity of different cell proliferation phases also hinders the microscopic analysis of its cell size distribution [24]. The Coulter counter is a different electronic counting method that was developed for counting blood cells. However, the technology has also been widely used for counting microalgal cells [25,26,27,28]. This electro-sensing area approach measures the impedance pulses produced by particles suspended in an electrolyte solution while passing through a small pore in a glass tube [29,30]. The proportional relationship between the impedance pulses and particle volume provides instrumental information about the particle volumes and numbers with high intrinsic precision based on large counting numbers.

Recently, a new microalgal strain was isolated accidentally in our laboratory when we isolated bacteria from the biofilms growing on microplastic samples [31]. This algal strain grows rapidly on bacterial culture medium and grows under light incidence, suggesting that this algal strain has the potential to perform auto- and heterotrophic growth. In this study, we first identified this algal strain based on its 18S rRNA-ITS1-5.8S rRNA-ITS2 operon sequence. We then compared the growth performance of this algal strain under auto-, hetero-, and mixotrophic conditions. Additionally, we applied a Beckman-Coulter Multisizer 4e analyzer to monitor the change in cell size of this algal strain under these three different growth modes. The algal strain was identified as *A. protothecoides* AS-1. Interestingly, different cell size distribution patterns were observed for this alga when it was cultivated under auto-, hetero-, and mixotrophic conditions, respectively.

## 2. Materials and Methods

### 2.1. Isolation of Green Algal Strain AS-1

**Figure 1:**
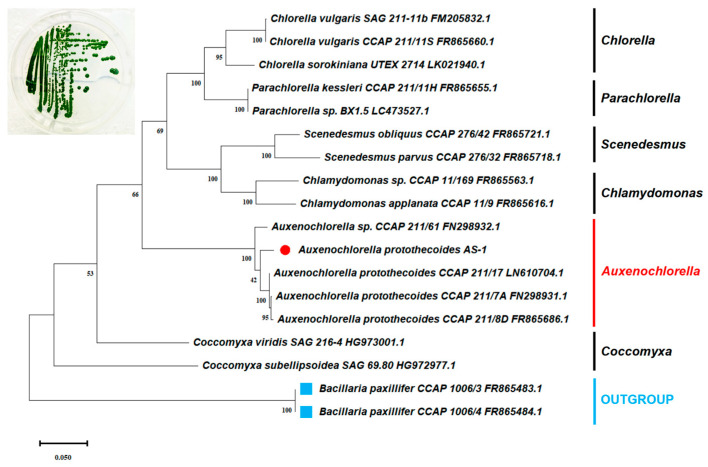
The phylogenetic relationship between strain AS-1 and other representatives of microalgae based on the sequences of the 18S rRNA-ITS1-5.8S rRNA-ITS2 operon. The tree was generated by the maximum likelihood method with 100 bootstrap replicates. The figure on the upper left shows AS-1 colonies on an R2B agar plate. R2B is a bacterial culture medium but AS-1 proliferates quickly on the R2B medium.

Strain AS-1 was isolated from microplastic beads incubated in the Baltimore Inner Harbor (39°17′11.05″ N, 76°36′22.77″ W) during an incubation study of microplastics [31]. Microplastic beads with green spots can be seen after the incubation (Figure S2). The original intention was to isolate heterotrophic bacteria from microplastic biofilm [31]. A dark green colony appeared quickly upon inoculation and was purified multiple times on the R2B agar plate (Figure 1). The isolate was initially considered as bacteria as it grew on the heterotrophic medium. It was named strain AS-1 because Ana Sosa first isolated this strain. 

### 2.2. Identification of Strain AS-1 Based on the Sequence of the 18S rRNA-ITS1-5.8S rRNA-ITS2 Operon

The sequence of the 18S rRNA-ITS1-5.8S rRNA-ITS2 operon was recovered when the partial genome of AS-1 was sequenced. The total DNA of AS-1 was extracted using a modified phenol–chloroform protocol [32]. The purity and quantity of the extracted DNA were assessed with a NanoDrop Spectrophotometer ND-1000 (ThermoFisher Scientific, Inc., Waltham, MA, USA. The genome sequence was determined using the New-Generation Illumina MiSeq (Illumina, Inc., San Diego, CA, USA) at the BioAnalytical Services Laboratory at the Institute of Marine and Environmental Technology. This sequencing effort was mainly to sequence the chloroplast genome of AS-1, not the chromosomal genome of AS-1. Genome data were assembled using SPAdes (Version 3.15.4). The 18S rRNA-ITS1-5.8S rRNA-ITS2 region of AS-1 was identified from the genome data using the 18S rRNA-ITS1-5.8S rRNA-ITS2 operon of *A. protothecoides* SAG 211/8D (Accession number: FR865686.1). The sequence of the 18S rRNA-ITS1-5.8S rRNA-ITS2 operon of AS-1 was aligned using MEGA 11 with the available data from the public NCBI nr database based on the BLAST result. This operon sequence of AS-1 was deposited in the NCBI database under accession number PP623876. Sequences were trimmed and aligned using the ClustalW method with MEGA 11. Maximum likelihood trees were constructed with 100 bootstrap values. The reference sequences used for the phylogenetic tree construction were retrieved from the NCBI nr database, and the corresponding accession numbers were included.

### 2.3. Growth of A. protothecoides AS-1 under Different Trophic Modes

The autotrophic, heterotrophic, and mixotrophic growth of *A. protothecoides* AS-1 was tested in BBM (Bold Basal Medium, pH = 7), TSB (Tryptic Soy Broth, pH = 7), and BBM+TSB (pH = 7), respectively. BBM+TSB was prepared using BBM as a base to prepare TSB (weighting and adding TSB ingredients to the BBM liquid culture medium). Experiments were carried out with batch culture in 125 mL baffled flasks. The BBM medium consisted of the following (g/L): Boric Acid, 11.42; Manganese Chloride • 4H_2_O, 1.44; Calcium Chloride, Anhydrous, 18.87; Potassium Hydroxide, 31.0; Cobalt Nitrate • 6H_2_O, 0.49; Potassium Phosphate, Dibasic, 75.0; Cupric Sulfate • 5H_2_O, 1.57; Potassium Phosphate, Monobasic, 175.0; EDTA, Disodium Salt, 63.61; Sodium Chloride, 25.0; Ferrous Sulfate • 7H_2_O, 4.98; Sodium Molybdate, 1.19; Magnesium Sulfate, Anhydrous, 36.63; Sodium Nitrate, 250.0; Zinc Sulfate • 7H_2_O, 8.82. The TSB medium consisted of the following (g/L): Tryptone, 17.0; Soytone, 3.0; Dextrose, 2.5; Sodium Chloride, 5.0; Dipotassium Phosphate, 2.5.

A colony of AS-1 from an agar plate was inoculated in 125 mL baffled flasks with 50 mL BBM+TSB medium, and the culture was grown mixotrophically for three days. After three days of acclimation, 2.5 mL of algal culture was pipetted into each flask containing 50 mL of medium (at about a 5% inoculation rate). The initial Optical Density (OD) at 680 nm was kept at 0.3–0.5 [33]. Flasks with culture were placed in a temperature-controlled incubator (Eppendorf, Inc., Hamburg, Germany) with a shaking speed of 120 rpm, temperature setting at 25 °C, and under continuous white LED lighting at 30 μmol m^−2^ s^−1^. Auto- and mixotrophic cultures were grown under a 16 h light and 8 h dark regime, while heterotrophic cultures were grown in the dark. The culture flasks were sealed with breathable membranes with pores (0.2–0.3 μm) to avoid potential contamination from the air. 

### 2.4. Growth Measurement

Cell growth was monitored by Optical Density (OD) measurement, cell counting, and dry biomass. Optical Density (OD) at 680 nm was measured using spectrophotometry DU800 (Beckman Coulter, Brea, CA, USA). Cell counts were performed using the Coulter counter 4e (Beckman Coulter). The filtration method was employed for cell dry weight measurement. 5 mL of the sample was filtered through a pre-weighed glass microfiber filter paper (Whatman GF/C, 1.2 μm) and washed twice with 5 mL of distilled water. The biomass retained on the filter paper was dried overnight at 100 °C. After cooling, the biomass dry weight was calculated based on the weight difference before and after filtration. For cell size measurement, 4 mL of samples were collected, fixed with 0.5% glutaraldehyde, and stored at 4 °C.

### 2.5. Observation of Algal Cells with Light and Fluorescence Microscope

We observed the algal cells using a Zeiss Axioplan microscope under a 100× oil lens (Oberkochen, Germany), with either light or epifluorescence mode. We also applied SYBR Green I stain to visualize the algal nuclei. To stain the algal cells, 5 mL of 1% (*v*/*v*) SYBR Green I in TBE buffer (mmol/L: boric acid 90, Tris 40; EDTA 2; pH 7.6) was added to a 10 mL cell suspension in culture solution; the mixture was incubated in darkness at room temperature for two hours [34]. Slides were allowed to stabilize for 10 min before observation. Then, filter set 49 (G365 for the excitation filter, FT395 for the dichroic mirror, BP450/50 for the emission filter) was used for observation. For light microscope observation, twenty algal cells per sample were captured randomly using the ZEN 2012 software (Zeiss Inc., 2013), and their cell sizes were measured. The cell size was calibrated with a micrometer (American Optical Company, Inc., Buffalo, NY, USA). The average cell diameter with standard deviation was calculated for each sample (See Supplementary Figure S1).

### 2.6. Cell Size Measurement with the Coulter Counter

The cell size was measured with a Beckman Coulter Multisizer 4e analyzer using 0.2 mm filtered isotonic II (Beckman Coulter) as the diluent and blank. The samples were fixed with 0.5% glutaraldehyde and were diluted 10,000 times with filtered IsoFlow Sheath Fluid (Beckman Coulter), and 500 μL of algal culture was analyzed each time. Particle size distribution was obtained with a 70 μm aperture, which measured particle size ranging from 1.4 to 56 μm. Data on cell diameter and density of a peak were obtained with the software Multisizer v4.03 (Beckman Coulter). Raw data sets were retrieved for plotting size distribution patterns.

### 2.7. Data and Statistical Analysis

The growth data of the algae were processed with GraphPad Prism 9.0 (GraphPad Software Inc., San Diego, CA, USA) or Excel v2.73 to generate growth curves. Data in triplicates were expressed as mean ± standard deviation. Student’s *t*-test and one-way ANOVA were used in the post hoc analysis of this study. The results were considered statistically significant if *p* < 0.05.

## 3. Results

When we tried to isolate heterotrophic bacteria from the Baltimore Inner Harbor using bacterial culture medium R2B, a large colony with a dark green color was formed on the R2B plate. This green isolate, AS-1, also grew rapidly in the R2B liquid medium and appeared to be eukaryotic microalgae under the light microscope. The sequences of the 18S rRNA-ITS1-5.8S rRNA-ITS2 operon showed that AS-1 shares the highest sequence identity (~97%) with three different strains of *A. protothecoides* CCAP 211/17, CCAP 211-7a, and CCAP 211/8D). The phylogenetic analysis based on this operon further confirms that strain AS-1 is most closely related to several *A. protothecoides* strains (Figure 1). Therefore, we named the strain AS-1 as *A. protothecoides* AS-1. 

**Figure 2:**
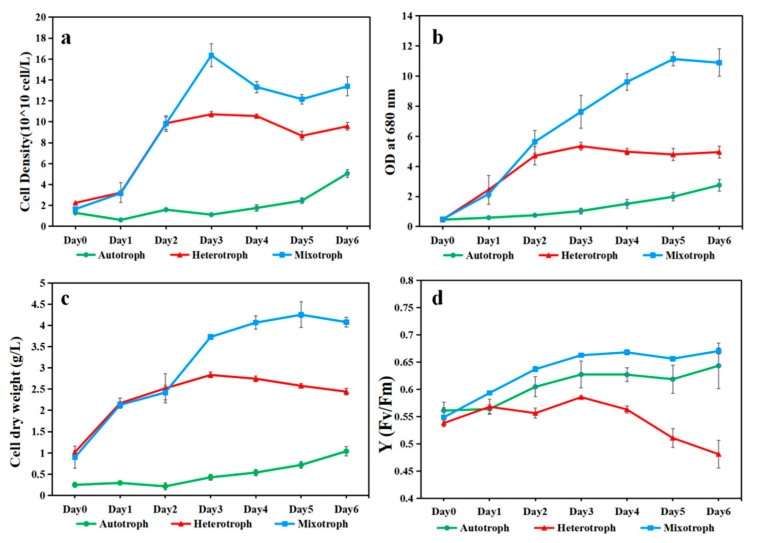
The growth performance of *A. protothecoides* AS-1 under auto-, hetero-, and mixotrophic growth conditions in 6 days. (**a**) Cell density of *A. protothecoides* AS-1 measured by the Beckman Coulter Counter. (**b**) Optical Density (OD) at 680 nm (**c**) Dry weight. (**d**) The fluorescence-based maximum quantum yield for photosystem II (Fv/Fm) of *A. protothecoides* AS-1.

*A. protothecoides* AS-1 grown under the mixotrophic condition yielded a higher cell density than those grown under the autotrophic and heterotrophic conditions, based on cell counts, OD680, and dry biomass data (Figure 2a–c). Autotrophic growth of AS-1 yielded the lowest cell density. In the first two days, the hetero- and mixotrophic growth of AS-1 appeared to have a similar quick start. However, after day 2, the mixotrophic growth outperformed the heterotrophic growth. AS-1 can reach more than 4 g/L biomass (dry weight) in 6 days (Figure 2c) and 1.6 × 10^8^ cells/mL on day 3 (Figure 2a). In terms of photosynthetic efficiency, the mixo- and autotrophic cultures shared a similar pattern, but the heterotrophic culture had a downward trend after day 3 (Figure 2d).

**Figure 3:**
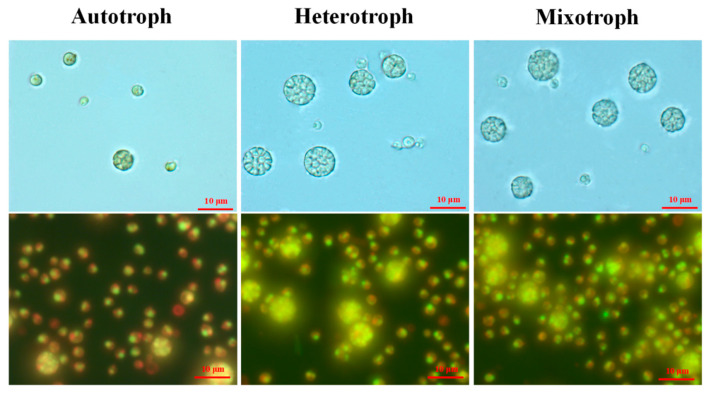
Microscopic images of *A. protothecoides* AS-1 under different trophic modes on day 1. The upper panel images were taken under a light microscope and the lower panel images were taken using epifluorescence microscopy. Algal cells were stained with SYBR Green I to better visualize the nuclei. The autofluorescence of chlorophyll yielded a red color. The scale bars (10 µm) in the upper panel images apply to all images here.

During the growth experiment, we observed a certain number of enlarged cells under all the growth modes (Figure 3). While the majority of AS-1 cells have cell sizes ranging from 3 to 5 µm in diameter, the size of the large cells ranges from 6 to 10 µm in diameter (Figure 3). The large cells seem to contain multiple small cells, a scenario similar to the relationship between mother and daughter cells (see the discussion). Up to 16 small cells within one large cell were observed. Thus, large cells are identified as mother cells and small cells as daughter cells (or autospores). It then became interesting to know how the number and cell size of the mother and daughter cells change at different growth stages and with different growth modes.

**Figure 4:**
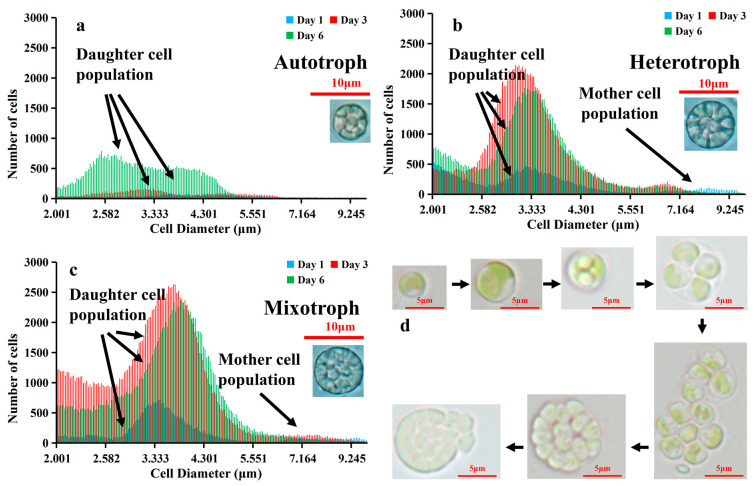
The size distribution pattern (2–10 µm) of *A. protothecoides* AS-1 on day 1, day 3, and day 6, measured by the Coulter counter. (**a**) Autotrophic condition; (**b**) Heterotrophic condition; (**c**) Mixotrophic condition; (**d**) Cell cycle of *Auxenochlorella protothecoides* AS-1.

To understand if different cell sizes occur in different trophic modes and how they change over incubation time, we used a Beckman Coulter counter to estimate the cell number and size of AS-1. Different cell size distribution patterns were observed for the auto-, hetero-, and mixotrophic modes (Figure 4a–c). We defined mother cells as those with cell diameters ranging from 6 to 10 μm and daughter cells from 2 to 6 μm. Under the autotrophic conditions, daughter cell populations had a wide cell size range (2–6 μm) with less distinguishable peaks (Figure 4a). In contrast, AS-1 daughter cells grown under the hetero- and mixotrophic conditions had an obvious peak at 3.5 μm. More mother cells (6–10 μm) under the hetero- and mixotrophic conditions were seen compared to the autotrophic condition (Figure 4a–c). Peak heights and positions changed with incubation days and trophic modes, suggesting that the cell size of AS-1 varies with nutrient sources and growth time. 

**Figure 5:**
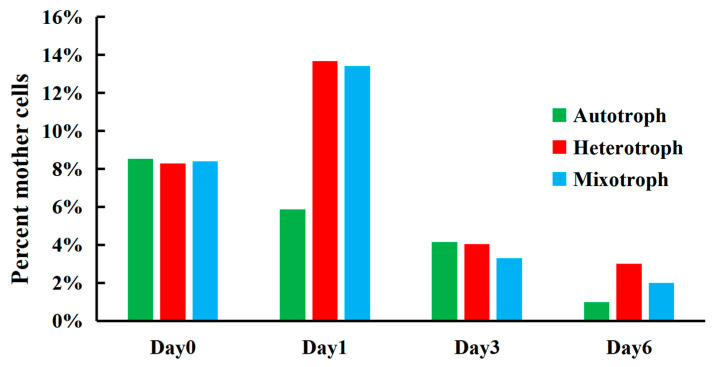
Percentage of mother cells (6–10 μm) in three different trophic modes on day 0, 1, 3 and 6.

We further calculated the contribution of mother cells to total cell density for the three different trophic modes over time. At the beginning of the experiment (day 0), mother cells contributed to 8% of the total cell counts (Figure 5). The percentage of mother cells increased rapidly (up to 14%) on day 2 for heterotrophic and mixotrophic AS-1, while the percentage of mother cells decreased for autotrophic AS-1 on day 1. On day 3 and 6, the percentage of mother cells decreased gradually and reached the lowest points for all three growth modes on day 6 (Figure 5). 

## 4. Discussion

The algal isolate was identified as *Auxenochlorella protothecoides*, strain AS-1. *A. protothecoides* (formerly known as *Chlorella protothecoides*) belongs to the family *Chlorellaceae* [35]. The subgenus *Auxenochlorella* within genus *Chlorella* was created by Shihira and Krauss [36] and later became a genus *Auxenochlorella protothecoides* (Krueger), according to Kalina and M. Puncoch [37]. Interestingly, *A. protothecoides* has the smallest chloroplast genome of photosynthetic green algae [38]. It is closely related to another pathogenic colorless alga, *Prototheca wickerhamii*, that lives a parasitic life [39]. Our phylogenetic tree based on the 18S rRNA-ITS1-5.8S rRNA-ITS2 sequence supports AS-1 to be classified as *A. protothecoides*. *A. protothecoides* is a facultative heterotrophic microalga and has been considered for use in biodiesel production as this strain grows fast under heterotrophic conditions [40,41,42]. In addition, *Auxenochlorella* species have been used to treat sewage because they are efficient in removing organic carbon and nitrogen, and inorganic nutrients [43,44,45]. 

AS-1 achieved high mixotrophic biomass (>4 g/L) when supplemented with 2.5 g/L glucose. It has been known that a mixotrophic cultivation strategy can achieve higher biomass [43,46,47,48,49,50]. Heredia-Arroyo et al. [51] used *C. protothecoides* 249 to achieve high mixotrophic biomass (>4 g/L) with 15 g/L glucose but with no significant difference with heterotrophic growth. *A. protothecoides* SAG 211-7a also achieved similar biomass (>4 g/L) on mixotrophic conditions with 10 g/L glucose [52]. *A. protothecoides* can use organic carbon efficiently [53]. Our results showed that under the mixotrophic mode, AS-1 used less glucose (2.5 g/L) to gain biomass over 4 g/L compared to earlier studies. This is probably due to the combined effect of different factors, including the strain of algae, the C/N ratio of the medium [54], and the possible impact of oxygen supply [55]. 

Giant cells appear more frequently under hetero- and mixotrophic conditions (Figure 3). Green algae have the following two fission modes: binary and multiple fission [11,56]. A classic mother cell undergoing multiple fission (*Chlorella ellipsoidea* and *Chlamydomonas*) is a multi-nuclear cell similar to a ‘cluster’ [11,57]. Therefore, the division pattern of AS-1 could be similar to that of *Chlorella* and *Chlamydomonas* [11,58]. It has been reported that *A. protothecoides* growing in both autotrophic and heterotrophic conditions showed the same division pattern [59]. The increased growth rates can lead to the overlapping division sequences of multiple fission, resulting in a giant mother cell with more than 2^n^ daughter cells [59,60]. Therefore, higher growth rates of AS-1 in the hetero- and mixotrophic conditions lead to more giant AS-1 mother cells compared to the autotrophic condition (Figure 3). Earlier studies showed that *A. protothecoides* cells under the heterotrophic growth are filled with lipid vesicles (more than 50% dry weight) [40,61]. Thus, the enlargement of AS-1 cells can also partially be attributed to lipid vesicles. 

The size distribution patterns of *A. protothecoides* AS-1 grown under the heterotrophic and mixotrophic modes are similar to each other, but they are very different from AS-1 grown under the autotrophic mode (Figure 4). The cell size of microalgae can be influenced by the process of cell division [13,28,56]. The change in algal cell size under different trophic modes has been previously reported. Chioccioli et al. [62] observed larger *Chlorella vulgaris* CCAP 211/11B cells in the culture media supplemented with glucose. Based on the flow cytometry analysis, Sánchez-Alvarez et al. [63] found that *Marinichlorella kaistiae* KAS603 preferred maintaining mother cells under nutrient-rich conditions and daughter cells under nutrient-poor conditions. Based on their microscopic observation, Li et al. [64] noticed that cells of mixotrophic *Asterarcys* sp. were larger than those under autotrophic conditions. Understanding cell size variation during the growth phase is important because cell sizes affect the nutrient uptake efficiency of algae. Nutrient deprivation conditions seem to favor the production of small cells as a higher surface-to-volume ratio performs better with nutrient absorption [63,65], while large cells can store nutrients for more extended periods due to higher nutrient storage capacity [63,66]. Therefore, different population structures can be observed in different nutritional environments [58]. We observed that AS-1 grown under the hetero- and mixotrophic conditions had the highest percentage of mother cells on the second day, and the proportion decreased in the following days (Figure 5). The reduced number of mother cells is likely caused by decreased nutrient concentration. The decrease in AS-1 mother cells under autotrophic growth conditions could be related to the slow growth of AS-1. The high percentage of mother cells on day 0 is due to the amount of mother cells carried from the seed culture. 

The Coulter counter enabled us to enumerate algal cells with their size information. A large number of cells with different size ranges can be analyzed quickly with the Coulter counter, but it does not provide cell images like the microscopic method. On the other hand, counting cells and measuring cell size using a microscope can be tedious and time-consuming. To ensure that the cell size measurement based on the Coulter counter is accurate, we also measured the cell size using the microscopic method. The average diameter of cells under the microscope is consistent with the average cell size measured by the Coulter counter (Figure S1), suggesting that both methods are comparable. Microscopic results also suggested that some binary fission cells share a similar size to some large undivided single cells (See Figure 3). Therefore, classifying algal cells into different stages based on cell size may be biologically inaccurate [11]. To better understand the cell division (i.e., mother cells vs. daughter cells) or cell morphology of microorganisms, the traditional microscopic observation still has its value. 

## 5. Conclusions

In this study, we compared the growth performance of our newly isolated microalga, *A. protothecoides* AS-1, under autotrophic, heterotrophic, and mixotrophic conditions. AS-1 can achieve high biomass and cell density quickly under mixotrophic growth. We noticed that AS-1 maintained slow growth under autotrophic conditions. Cells of AS-1 with different sizes were observed under different trophic modes under the microscope. Some mother cells can contain up to 16 daughter cells. The fast growth of AS-1 caused cell enlargement (mother cells), resulting in more mother cells in the mixotrophic and heterotrophic cultures on day 2. The reduction in mother cells in the later growth stage could be related to the slow growth limited by nutrients. We provided a comprehensive study on the growth performance and cell size variation of *A. protothecoides* AS-1 under autotrophic, heterotrophic, and mixotrophic growth conditions. *A. protothecoides* is known to have great potential to be used for wastewater treatment and the development of biofuels and other valuable products. We will continue to explore the industrial use of this new strain of *A. protothecoides*.

## Data Availability

Data are contained within the article and Supplementary Materials.

## Supplementary Materials

The following supporting information can be downloaded at: https://www.mdpi.com/article/10.3390/microorganisms12040835/s1, Figure S1: A comparison of the average cell diameter obtained from the Coulter counter Multisizer 4e with data from the light microscope (Based on 20 cells). Figure S2: The biofilm on microplastics under a light microscope. Greenish-colored biofilm was observed on the surface of microplastics.
