# Supplementary material for: Growth and Cell Size of Microalga Auxenochlorella protothecoides AS-1 under Different Trophic Modes
Source: Microorganisms. 2024 Apr 20;12(4):835. doi: 10.3390/microorganisms12040835 (PMC11052296; doi:10.3390/microorganisms12040835)
Supplement: Supplementary file 1 [file microorganisms-12-00835-s001.zip › microorganisms-2938989-supplementary.pdf]

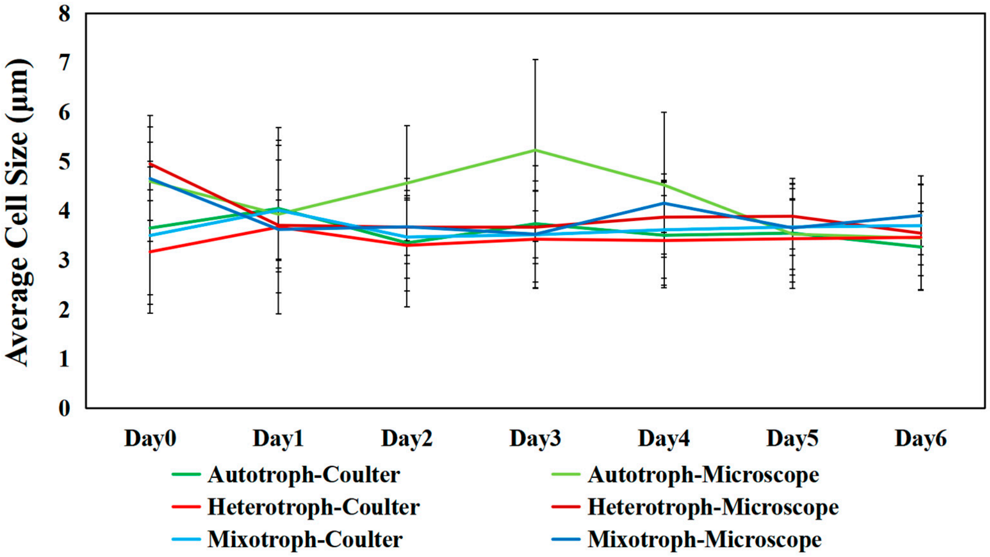

Figure S1. A comparison of the average cell diameter obtained from the Coulter counter Multisizer 4e with data from the light microscope (Based on 20 cells).

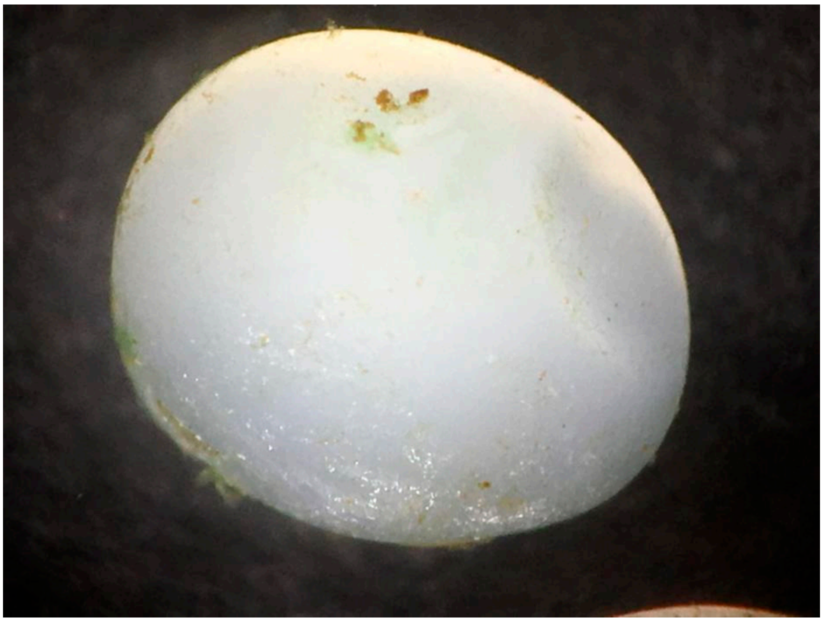

Figure S2. The biofilm on microplastics under a light microscope. Greenish-colored biofilm was observed on the surface of microplastics.
